# Monitoring Aquaculture Water Quality: Design of an Early Warning Sensor with *Aliivibrio fischeri* and Predictive Models

**DOI:** 10.3390/s18092848

**Published:** 2018-08-29

**Authors:** Luís F. B. A. da Silva, Zhaochu Yang, Nuno M. M. Pires, Tao Dong, Hans-Christian Teien, Trond Storebakken, Brit Salbu

**Affiliations:** 1 Institute of Applied Micro-Nano Science and Technology—IAMNST, Chongqing Key Laboratory of Colleges and Universities on Micro-Nano Systems Technology and Smart Transducing, Chongqing Engineering Laboratory for Detection, Control and Integrated System, National Research Base of Intelligent Manufacturing Service, Chongqing Technology and Business University, Nan’an District, Chongqing 400067, China; a61696@alunos.uminho.pt (L.F.B.A.d.S.); Nuno.Pires@usn.no (N.M.M.P.); 2 Department of Microsystems—IMS, Faculty of Technology, Natural Sciences and Maritime Sciences, University of South-Eastern Norway, Postboks 235, 3603 Kongsberg, Norway; 3 Centre for Environmental Radioactivity (CERAD CoE), Norwegian University of Life Sciences (NMBU), Faculty of Environmental Sciences and Natural Resource Management, P.O. Box 5003, NO-1432 Ås, Norway; hans-christian.teien@nmbu.no (H.-C.T.); brit.salbu@nmbu.no (B.S.); 4 Faculty of Biosciences, Department of Animal and Aquacultural Sciences, Norwegian University of Life Sciences, P.O. Box 5003, N-1432 Ås, Norway; trond.storebakken@nmbu.no

**Keywords:** *Aliivibrio fischeri*, water quality monitoring, whole-cell biosensor, linear concentration addition, linear independent action, aquaculture

## Abstract

A novel toxicity-warning sensor for water quality monitoring in recirculating aquaculture systems (RAS) is presented. The design of the sensor system mainly comprises a whole-cell biosensor. *Aliivibrio fischeri*, a luminescent bacterium widely used in toxicity analysis, was tested for a mixture of known fish-health stressors, namely nitrite, un-ionized ammonia, copper, aluminum and zinc. Two toxicity predictive models were constructed. Correlation, root mean squared error, relative error and toxic behavior were analyzed. The linear concentration addition (LCA) model was found suitable to ally with a machine learning algorithm for prediction of toxic events, thanks to additive behavior near the limit concentrations for these stressors, with a root-mean-squared error (RMSE) of 0.0623, and a mean absolute error of 4%. The model was proved to have a smaller relative deviation than other methods described in the literature. Moreover, the design of a novel microfluidic chip for toxicity testing is also proposed, which is to be integrated in a fluidic system that functions as a bypass of the RAS tank to enable near-real time monitoring. This chip was tested with simulated samples of RAS water spiked with zinc, with an EC50 of 6,46E-7 M. Future work will be extended to the analysis of other stressors with the novel chip.

## 1. Introduction

‘Toxicity’ stands for the grade of a substance or a mixture to cause a harmful effect to an organism. The maximum exponent of that effect is the death of the organism. On the other hand, at lower exposure doses, effects such as stress can be observed. Substances that induce stress are called stressors. In fish, stress can have consequences such as inhibited growth, weaker immune system, and behavioral changes [1,2]. The first two effects are very undesirable in the context of aquaculture, where optimal growth and low mortality rates are demanded to maximize profit.

Recirculating aquaculture systems (RAS) present an environmentally-friendly approach to aquaculture, as less water is wasted through recirculation, and nitrogen rich waste can be used as a fertilizer for aquaponics and copeponics [3]. Mortality rate related to pathogenic diseases are decreased by culturing the fish inland in tanks, using quality-controlled water systems. Trace metals such as zinc and copper can be supplied by feed [2], or instead by erosion of materials present in the RAS system and the water source itself, such as aluminum [4,5]. Other sources of stressors are excretion products by fish and uneaten feed as the case of un-ionized-ammonia or byproducts of its nitrification, as in the case of nitrite, can cause death by hypoxia via binding with the heme group of fish hemoglobin and modify it, thus preventing it to link to oxygen [6] Un-ionized ammonia, on the other hand, interacts with the fish nervous system [2]. Heavy metals are toxic to fish, interfering in osmoregulation and even leading to the accumulation of other toxic substances such as ammonia. Thus, it is of great importance to monitor the water quality parameters in RAS, particularly these stressors.

Nowadays, electrochemical analysis is often used to monitor RAS water quality. Typical parameters include temperature, salinity, pH, dissolved oxygen, or biological oxygen demand for oxygen, total dissolved solids or total organic carbon for detecting organic matter, ions and dissolved carbon, and oxygen reduction potential for alkalinity [4,7,8]. The electrochemical analysis is often performed by commercial sensors—e.g., Oxygard Pacific (OxyGuard International A/S, Denmark), Pentair Point Four (Pentair, UK). With these sensors, most of the measurements are not specific, giving a rough indication of water quality. More specialized tests for chemical identification and quantification still need to be done by sampling and analysis in the laboratory. Therefore, real-time monitoring of chemical stressors is still difficult. Moreover, those sensors do not take into account interactions between stressors, which can promote the toxicity of one another when combined.

In summary, real-time or near real-time monitoring of RAS water for chemical stressors—such as un-ionized ammonia, nitrite, aluminum, copper, and zinc—and their combined toxicity is required. Toxicity tests usually involve the use of model animals, algae, or microorganisms. Whole-cell toxicity biosensors provide a promising solution to miniaturized sensors, as their small size enables the incorporation into lab-on-a-chip platforms or field deployable sensors. *Aliivibrio fischeri*, a marine bacterium, has been used as a toxicity biosensor due to its high broad range sensitivity [9]. Its use as a water quality monitoring sensor has been proved in the commercially available Microtox CTM and TOXControl analyzers, as well as on industry effluents studies [10]. Its assay consists in the measurement of the inhibition of luminescence, produced by a chain of enzymatic reactions, which are known to be interfered by substances as antibiotics [11] or heavy metals [12]. The luminescence can be recorded using CCD cameras or photomultiplier tubes, as shown in work conducted in [13,14].

Disposable chips to identify toxic chemicals or general toxicity testing by using immobilized bioluminescent bacteria have been reported [13,15,16,17], either by using genetically modified bacteria or mammalian cells [15], immobilizing *Aliivibrio fischeri* in chip for heavy metal monitoring on environmental waters [16] or chemicals, such as naphthalene [18]. Zhao and Dong presented a microfluidic chip for drinking water quality monitoring [14], mixing both *Aliivibrio fischeri* and the water sample and subsequent measurement of the luminescence in a microfluidic chamber 20 min later. The inhibition was determined by normalization with the luminescence recorded using a control. This sensor could perform acute toxicity tests, being suitable to determine the toxicity of zinc, potassium dichromate, and copper.

Predicting highly toxic episodes can be as important as to detect them, enabling the application of counter-measures to keep a healthy fish culture. Therefore, there is an interest in the construction of prediction models to prevent highly toxic events. The available models are mostly applied to the field of drug and chemical discovery. Quantitative structure–activity relationship (QSAR) models have been used to predict the toxicity of new chemical and drug compounds, by comparing the similarity among chemicals with known chemical structures [19]. Binary decision models have also been developed [19]. Both types of models are easy to implement, but without considering possible interactions between the different compounds; alerting only to the presence/absence of such compounds and errors can be induced if the new chemical has a different mode of action. Dose–response or time–response models present a simple way of characterizing mixtures when the modes of action of the components in a mixture are unknown [19]. As the name implies, different doses of sample are used, and the corresponding response or effect is measured. Then a relation is drawn through different linear fitting models, such as Probit, or non-linear fitting—e.g., the Logit model. The dose can be expressed as a concentration or logarithm of a concentration that causes a percentage of an effect, named effect concentration (EC). Zadorozhnaya et al. [20] compared the Microtox^®^ assay with an array of electrochemical sensors conjugated with a prediction model of single chemical toxicities as well as freshwater samples based on random forest, principal component analysis, and random KNN. The sensor array behaved poorly, with relative errors between 20% and 25%. Hence, the use of *Aliivibrio fischeri* allied with prediction models may constitute a better alternative to electrochemical sensors for sample toxicity assessment.

When in mixture, the toxicity of compounds can express different behavior: as stated by Bliss [9], mixtures can behave additively, synergistically or antagonistically. Most mixtures are considered to behave additively. Two classical models have been proposed, based on the mode of action of components of the mixture: concentration addition (CA), where all components have the same mode of action, and the independent action model (IA), where the different components have dissimilar mode of action [9]. More recently, new models with enhanced accuracy have been reported in the literature, such as the generalized concentration model proposed by Tada [21], as well as linear regression models including linear concentration addition and linear independent action (LIA) models presented by Qin et al. [22].

This work presents a novel concept of sensor for water quality monitoring in RAS by demonstrating a whole-cell biosensor conjugated with toxicity prediction models. Furthermore, the design of an integrated microfluidic chip to test water toxicity in RAS is also presented and its usability was studied in simulated conditions.

The following sections describe and analyze the work done. Section 2 starts by describing the material and methods employed in the design of the experiments for the prediction models and the predicates for the design of the microfluidic chip. In Section 3, the results are presented and discussed using the same structure as Section 2. Lastly, the main conclusions are drawn in Section 4.

## 2. Material and Methods

Here we started from uniform design ray which is a method of planification of toxicity studies that reduces the amount of experiments needed to characterize a mixture of toxicants. It is followed by the material and methods employed for the study, such as reagents, equipment, and protocols used for the study of the mixture and previous information related to the study of the stressors when single in solution. The prediction models are explained in the data processing part, along with the statistical methods and software employed for this goal. Finally, it is introduced the assumptions for the design of a lab-on-a-chip system for toxicity testing.

### 2.1. Uniform Ray Design Method

The experiment was designed by following the uniform design ray (UD) presented by Liu et al. [23]. This method combines the logic of the fixed ratio ray design with the uniform design, reducing the number of experiments required to characterize the toxicity response of an organism to a mixture of toxicants. It consists of the creation of a uniform distribution of effect concentrations per experiment. This uniform distribution is achieved based on the number of concentrations and the number of components of the mixture and the number of factors s, which regulates the maximum number of experiments needed. Each experiment forms a ray, by creating a dilution series from the selected concentrations. Thus, having a mixture with five components, a uniform table U_7_ (7^6^) was designed (Table S1 of the Supplementary Material).

### 2.2. Reagents and Equipment

The Lumoplate Ultimate Matrix kit (EBPI, Mississauga, ON, Canada), based on freeze-dried *A. fischeri* as a test organism for toxicity, was used in our study. Stock solutions of ammonium chloride (Sigma-Aldrich, Oslo, Norway), zinc sulfate heptahydrate (Sigma-Aldrich, Oslo, Norway), aluminum chloride (Sigma-Aldrich, Oslo, Norway), and copper sulfate (Sigma-Aldrich, Oslo, Norway) were prepared in deionized water. Concentrations of ammonia and zinc were confirmed using the salicylate method and the zinc test method (photometric 0.05–2.50 mg/L Zn spectroquant from Merck Milipore). The nitrite ion standard solution 1 g/L (Merck Milipore, Beijing, China) was used to prepare the nitrite stock solution. The luminescence was recorded with the Synergy 2 microplate reader (BioTek, Winooski, VT, USA) in a 96 well microplate. A thermo-block was employed to keep the temperature of the microplate constant at 15 °C and the samples were kept at constant temperature at 15 °C in a Lauda CP6 thermo-bath.

### 2.3. Single Stressors Study

In order to analyze the behavior of toxicants in a mixture, the toxicity of individual toxicants to *A. fischeri* needed to be studied. Hence, toxicity tests for all the toxicants were in advance conducted based on their typical concentration in Atlantic salmon RAS and their safe limits to this fish. Tables S2–S4 of the Supplementary Material present the concentrations used for copper, aluminum, and zinc.

### 2.4. Mixture Study: Preparation and Test

For the evaluation of the mixture of nitrite, un-ionized ammonia, copper, aluminum, and zinc, each effect concentration of each component in the mixture was calculated according to their respective dose–response curves. Table 1 shows the concentrations of each ray.

For both tests, based on the manufacturer’s protocol, freeze-dried bacteria were reactivated by quickly pouring a vial of a saline solution (reagent diluent) in a freeze-dried bacteria vial and stored in the freezer at 4 °C for 30 min.

The mixtures and controls were prepared and pipetted into 1.5 mL centrifugal tubes. To mimic typical Norwegian RAS water, soft EPA artificial water was employed as an initial matrix. Then, to stabilize the reading since a marine species is being used, this water was mixed with 10% volume of osmotic adjustment solution (OAS), a saline solution provided in the Lumoplate Ultimate Matrix kit, to a final salinity of 2%.

For both experiments, 400 µL of sample, 300 µL of zinc control and 1.5 mL of sample diluent, another saline solution similar to reagent diluent, were used. 

The mixture samples were prepared by pipetting the correct volume of each stressor into the corresponding tube to achieve the specific concentration described in Table I already complemented with the mixture of EPA water and OAS solution to a total of 400 µL.

A dilution series of 2, 4, 8, 16, 32, and 64 was created for each mixture ray directly in the microplate for the mixture tests. As for the single tests, each concentration was prepared and pipetted in each tube. The tubes and the bacterial vial were put into to the thermo-bath and maintained at 15 °C. Also, the microplate was maintained at 15 °C in the thermo-block prior to the test. After 30 min, 100 µL of bacterial solution were pipetted in each well of the microplate followed by 100 µL of mixture solution and sample diluent in their respective wells, for the single stressors test. For both tests, sample diluent was used as a negative control. As for the mixture tests, 100 µL of sample diluent was pipetted in all wells, except for the most concentrated solution of zinc and samples row, followed by 200 µL of the zinc solution and sample was pipetted in a micro-well with only the bacteria solution. The luminescence was recorded two times, first at the moment of addition of the sample and then 5 min after contact, being the microplate maintained at a constant temperature of 15 °C by incubation in the thermo-block.

### 2.5. Data Processing

Microsoft Excel 2016 was used to calculate the observed inhibition, the total concentration of the mix according to concentration addition model and the expected inhibition according to the independent action model. The observed inhibition is calculated based on normalized decrease of bioluminescence, taking into account the initial luminescence, in percentage. The dose–response curves for the experimental data were fitted using a four parameter inverse logistic function following Equation (1) [24]

(1) $$y=A_{L}+\frac{(A_{H}-A_{L})\;}{1+10^{\log(x_{c}-x)\times p}}$$

where *y* stands for inhibition and *x*, *x_c_* are the effect and central concentrations, respectively. The central concentration can be defined as the concentration of the half response. *A_H_* is the upper inhibition limit and *A_L_* the lower limit and *p* is the slope. Concentrations *x* are computed as the logarithm of the concentration.

Each effect concentration can be calculated according to Equation (2) [24]

(2) $$Log(ECx)=\log(xc)+\frac{\log(\frac{x}{100-x\;})}{p}$$

#### 2.5.1. Toxicity Linear Models for the Single Stressors Study

The linear independent action and linear concentration models were first described by Qin et al. [22]. They rely on an initial prediction of toxicity using the CA and IA models, which are then linearized using simple linear regression to predict the effective concentrations of the mixture or their inhibition. Therefore, first each mixture concentration was predicted according to the CA model using Equation (3)

(3) $$ECx_{mix\;}={\left(\sum_{i=1}^{n}\frac{p_{i}}{ECx_{i}}\right)}^{-1}$$

in which *p_i_* is the proportion of the *i*th component in the mixture, *ECx_mix_* is the predicted inhibition effect of the mixture, and *ECx_i_* is the expected concentration of the *i*th component *i* causing such inhibition. The expected inhibition caused by a certain concentration in the IA model was calculated according to Equation (4)

(4) $$E\%=1-\prod_{i=1\;}^{n}(1-F_{i}(p_{i}\times (ECx_{mix})))$$

where *E*% is the inhibition, in percentage and *F_i_* is the dose–response curve equation of the *i*th component that explains its toxicity, when in single. The predicted concentrations for CA were converted to a logarithmic scale and simple linear regression was applied to estimate parameters *b*_0_ and *b*_1_, according to Equation (5). For the LIA model, the individual concentrations of each component were estimated using their dose–response curve models and the predicted total concentration was calculated by the same principal using simple linear regression, according to Equation (6).

(5) $$pEC_{xLCA\;}=b_{0}+b_{1}\times pEC_{xCA}$$

(6) $$pEC_{xLIA\;}=b_{0}+b_{1}\times pEC_{xIA}$$

where *pEC_xLCA_* and *pEC_xLIA_* are the mixture effect concentrations, observed, and *pEC_xCA_* and *pEC_xIA_* are the predicted effect concentrations by the *CA* and *IA* models, in logarithm scale.

OriginPro 8 (Originlab Corporation, Northampton, MA, USA) was used to create the models using simple regression and plot it against the observed inhibition to create a dose–response curve, as well as to calculate the predicted EC50 through the model.

#### 2.5.2. Model Fitting and Statistical Evaluation

The observed data and the IA results were fitted by use of a four-parameter logit curve following the non-linear fit model in OriginPro8 (Originlab Corporation, Northampton, MA, USA), with inhibition of luminescence as a function of the logarithm of the total concentration of the mixture, in mM. Also, a 95% confidence interval of the fit was drawn. All the results were then plotted for comparison.

The model deviation ratio (MDR) [25] was calculated for LCA and IA models by the quotient of the expected EC50 of each ray and the observed EC50. Also, the relative deviations of EC50 predicted by each model were calculated.

### 2.6. Lab-On-A-Chip Study

In order to apply the assay in the field, a miniaturization of the assay and automation of the process would be necessary. One way to achieve this, is to design a microfluidic device to enable *A. fischeri* assays in lab-on-a-chip formats. 

As our goal was to create a field deployable system with low maintenance, and thus minimal human interference, disposable chip is not a solution. Our strategy was to design the reusable chip which may deploy and mix all the necessary reagents and samples.

Since the assay needs a high number of controls and reagents to be conducted in the microplate, an adaptation of the protocol had to be made. This was done to, on the one hand, maximize the ratio between the number of inlets and number of on-chip mixers, and on the other minimize the chip maintenance complexity. Hence, it was found suitable to have three inlets and two mixers: one for mixing the OAS solution and the RAS water sample and other to mix the resulting solution with the bacteria solution.

The chip is comprised of one measuring chamber where the bacteria would gather to maximize the bioluminescence. The bioluminescence is to be read by a photodetector such as a photomultiplier tube. Another chamber was used to evaluate the bacteria fitness for the test and normalize the luminescence recorded during the test after mixing the sample and the bacteria, or it could simply be used a two-time measurement, in which the luminescence of a negative control would be read prior to the test of the RAS water. 

The chip design was realized using Solidworks 2016 edition (Dassault Systems).

The chip was fabricated in a 3D printer (Objet30 Pro, Stratasys, Eden Prairie, MN, USA), by use of VeroWhitePlus, a patented white opaque material, which enables fast prototyping of microfluidic device. Besides, the material was chosen to minimize crosstalk luminescence between different structures of the chip, minimizing the noise in the measuring chamber.

Finally, the chip was tested with a dilution series of zinc, using three syringe pumps (Micro Syringe 1, Chemyx, Stafford, TX, USA) with a flow rate of 24, 21.6, and 2.4 µL/min for bacteria solution, sample, and OAS solution, respectively. The luminescence was recorded by a photosensor module H10722-110 (Hamamatsu, Japan) controlled by an Arduino Uno. The data were reduced as described in Section 2.5.

## 3. Results and Discussion

The results are presented and discussed in this section. Section 3.1 starts by presenting the results with single stressors. Section 3.2 follows by comparing the prediction ability of the two models and their suitability for use with a water quality sensor for RAS. Finally, Section 3.3 describes the results with the microfluidic chip and presenting a discussion.

### 3.1. Single Stressors Analysis

The resulting EC50 and curve parameters for the single stressors study can be seen in Table 2.

### 3.2. Comparison of LIA and LCA Models

Antagonistic relations are characterized by a decrease of the expected toxicity, as different stressors can exhibit different affinities to the same receptor, or complex in a less toxic chemical specie. Graphically, it can be identified by lower inhibition, as well as greater EC50 [26]. Conversely, synergetic relations cause a rise of toxicity, exhibiting higher inhibition at smaller concentrations.

**Figure 1:**
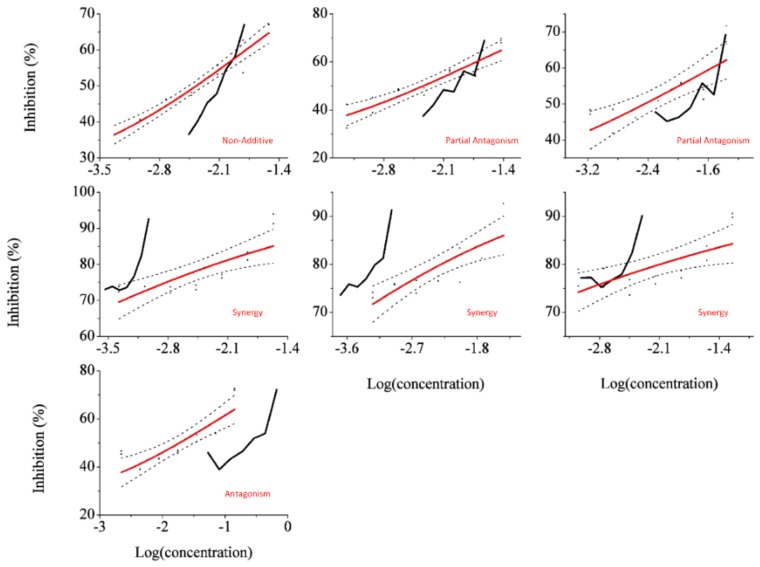
Dose–response curves for each ray, presenting inhibition in function of the logarithm of the total mixture molar concentration. The effect concentrations for each inhibition point were built according to the LIA model are presented in a black solid line. Observed inhibition points are displayed in black and were fitted in a four-parameter Logit function (red). 95% confidence limits of this fit are presented as the dashed lines.

As can be seen in Figure 1, in overall, the mixture presented non-additive behavior, according to the LIA model. Antagonism behavior was observed for Rays 1,2, 3, and 7 and synergism in the rest, which points to an inability to accurately predict the toxicity of this mixture.

**Figure 2:**
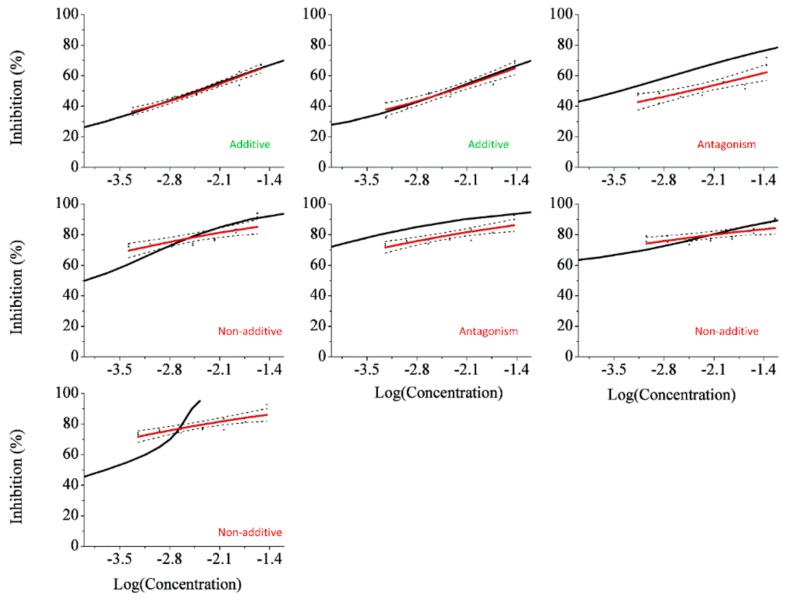
Dose–response curves for each ray, presenting inhibition in function of the logarithm of the total mixture molar concentration. Effect concentrations for each inhibition point were built according to the LCA model are presented in a black solid line. Observed inhibition points are displayed in black and were fitted in a four-parameter Logit function (red). 95% confidence limits of this fit are presented as the dashed lines.

As for the results with the LCA model, shown in Figure 2, it was observed additive behavior in Rays 1 and 2; antagonism for Ray 3 and 4; and partial synergism in Rays 3, 5, and 7. Nonetheless, it seems to have performed better than the LIA model. Observing the graphs for Ray 1 and 2, it seems as the model and the observed data curves almost superimpose.

Table 3 presents the constructed linear models, and their respective Pearson correlation coefficients.

It indicates that a better linear relationship between predicted values and mixture concentrations was achieved for LCA than LIA. We can see by the Pearson correlation coefficient that that Rays 4 and 6 were the hardest to model.

Table 4 presents the estimated 50% effect concentrations predicted by the two models and observed in the test, as well as the mean deviation error and the relative deviation of the models.

The MDR is an indicator of the behavior of a mixture. MDR values greater than the unity alert to antagonism, while values below 1 indicate synergism. The unity represents additive behavior [25]. This metric was chosen by its non-relation to one model, as well as its ease of use. According to the MDR values in Table 4, we may confirm the previous graphical analysis for the LIA. On the other side, for the LCA model, the graphical interpretation is harder, as we see partial antagonism and partial synergism in the same ray. This is disclaimed by the analysis of the MDR, which indicates antagonism behavior for Rays 3 to 7 for the LCA model.

Both presented high relative errors, being the LCA accurate for mixture Rays 1 and 2. This can be explained by the fact that both models rely on the principle of non-interaction between the components of a mixture [22]. Nevertheless, LCA seems to have a higher predictive value, with a mean absolute deviation of 4% and a root-mean-squared deviation error (RMSE) of 0.0623 against 31% and 0.735 for IA. Also, both CA and LCA are simpler to model, as CA relies on the known concentrations of the components of the mixture, while IA relies on point determinations based on the observed inhibition, implying a pre-fit, which induces higher error. Therefore, several experimental data points are needed to build an accurate model.

Compared with the work of Zadorozhnaya et al. [20], both algorithms in combination with the whole-cell biosensor outperforms on the relative deviation.

Due to the sensitivity to stressors and for being the most farmed fish in Norway, Atlantic salmon was chosen as our fish model. The recommended water quality limit values of the stressors being analyzed for Atlantic salmon are listed in Table 5.

Comparing these values with Table 1, we see that the concentrations used in this assay for all rays were above the limits in a RAS system for Atlantic salmon. Likewise, we can observe that the total concentration in each ray increases. Therefore, the LCA model is a valid model for predicting high toxicity events for Atlantic salmon’s RAS systems, with a relatively low relative deviation. Also, it describes additive behavior for lower concentrations, indicating that non-additive behavior may only occur when testing very high concentrations. This model, in conjunction with least-square regression could be used in a software to predict the evolution of toxicity in near real-time.

### 3.3. Microfluidic Chip for RAS

#### 3.3.1. Chip Design

**Figure 3:**
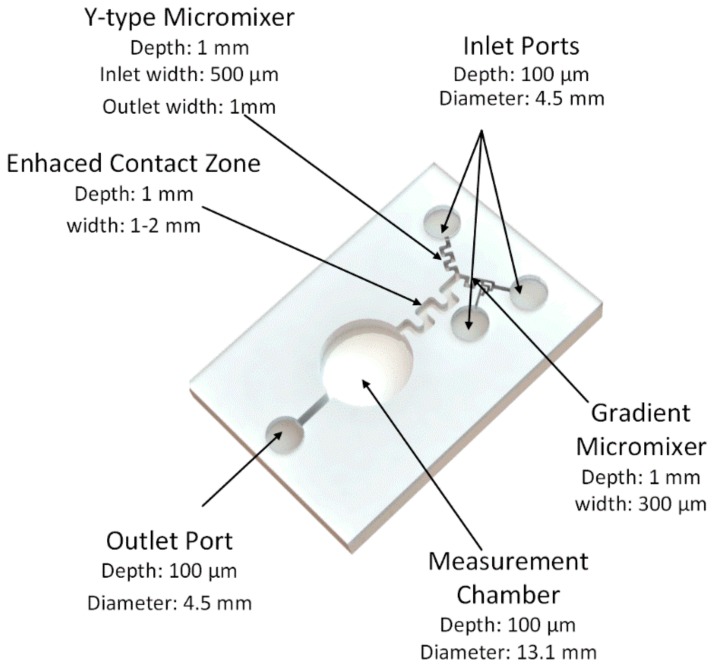
Lab-on-a-chip design. The chip was designed for water quality monitoring of Atlantic salmon RAS. The chip consists of three inlets, one inlet port, one measuring chamber, two micromixers, and an enhanced contact zone to improve interaction of the bacteria with the sample two serpentine channels to synchronize fluid mix.

As described in the Material and Methods section, three inlets, two micromixers, one measuring chamber, and one outlet were designed. The final design is presented in Figure 3. 

A measuring chamber with a final volume of 200 µL was designed in order to hold most of the bacteria in the final solution. All corners were filleted to facilitate post-test wash, promoting less dead volume and cell adhesion. 

Another chamber could be used to evaluate the bacteria fitness for the test and normalize the luminescence recorded during the test after mixing the sample and the bacteria, for example. This hypothesis was discarded as it implied a higher residual volume, which would increase unnecessarily the cost of the test. 

A y-type passive micromixer was used, as it presents as an efficient way of mixing two fluids. A 120° angle between the two inlet channels was chosen to enhance the mixing performance, as demonstrated by Shi et al. [28]. 

As for the second micromixer, it was designed due to the difference in flow rates between the OAS and sample solution injections, as the OAS should be 10% of the total volume of sample. This could create backflow of the OAS solution, hindering its mix with the sample, with a rise of sample toxicity and false positives as consequences. The original design was first presented by Mele [29] and consists on a branched channel design that enables a gradient mix by decreasing the pressure by half in each bifurcation. 

Two serpentine channels were then constructed ahead of the y-type micromixer to enable a synchronized arrival of sample and bacteria enabling a 1:1 ration mixture. The outlet of the micromixer had the minimum length, as it has been demonstrated that the maximum mixture occurs in the micromixer, independently of the length. Finally, an additional serpentine channel was used to reduce the flow velocity, enhancing the contact time. A residual volume of 40 µL was considered sufficient.

The proposed design is comparable in complexity to other microfluidic test platforms such as the one proposed by Bhalla et al. [30]. Their work demonstrated a two-step validation method for Adiponectin detection using ELISA. It presented a method of introducing multiple reagents that passively mix in *y*-type mixers and are pumped by multiple syringe pumps to a reaction chamber with a high aspect-ratio to increase interaction and consequent reading. However, in our case it needs not to introduce the reagents sequentially, the serpentine channels were used before mixing with the biosensor rather than introducing reagents by spaced y-type single branches.

**Figure 4:**
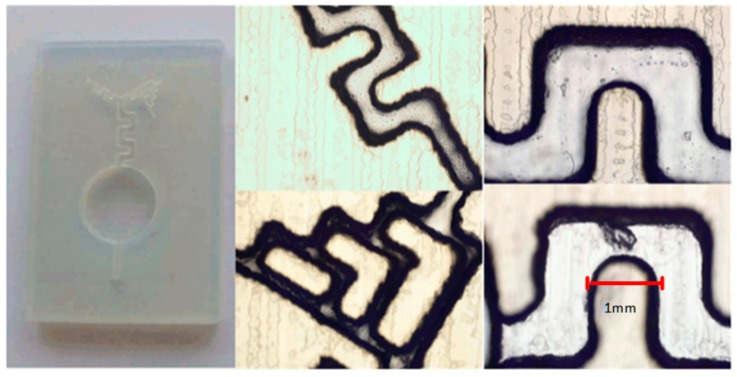
Fabricated chip in VeroWhitePlus. On the side are presented the serpentine channels and the gradient micromixers (photographs taken from an optical microscope at an 100× ampliation).

The fabricated chip is shown in Figure 4.

#### 3.3.2. Assay Protocol

The assay protocol is hereby presented and discussed. The whole process is to be controlled by a microfluidic control system through use of valves and pumps. The total time of the assay is 30 min, while reagents are loaded, the outlet is open, only getting close for the change of content in the syringe between the stages necessary to load and release of reagents and wash the chips. To compensate for the lack of one chamber, the assay is conducted in two steps. In the first step, a calibration for the background is made, with a negative control saline solution, introduced by the two reagent solutions in a total flow rate equal to the flow rate of the bacteria. After 5 min, when the measuring chamber is expected to be filled, bioluminescence is recorded with a photomultiplier tube. The fluids are released, and a wash step is done to remove used bacteria. Biofilm and stressors still present by flushing a saline solution through all inlets for enough time. Biofilm is formed by the release of inducers of bioluminescence. in a process named quorum sensing, Alternatively, a weak acid could be used [29], but would require an additional pump and add more complexity to the microfluidic control system.

**Figure 5:**
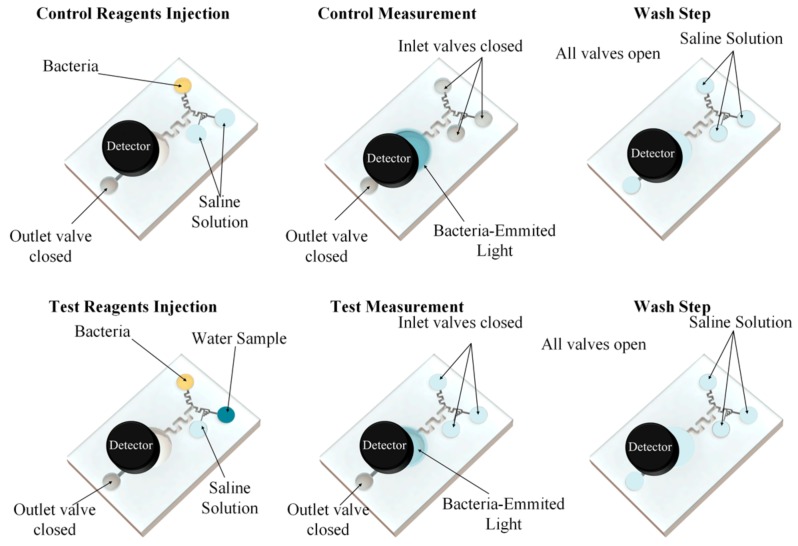
Visual schematic of the protocol. The test is conduct in two major steps: an initial calibration and posterior contact with the sample. A wash step is added between the two to wash used bacteria and toxic sample present that could interfere with the next measurement.

The signal would be sent to a microcontroller controlling the PMT and sent to a computer for further processing. A software to analyze the signals and calculate the inhibition, as well as to implement the prediction model and alert the fish farmer in case of present or future events. A visual schematic of the protocol is presented in Figure 5.

#### 3.3.3. Tests on Chip

**Figure 6:**
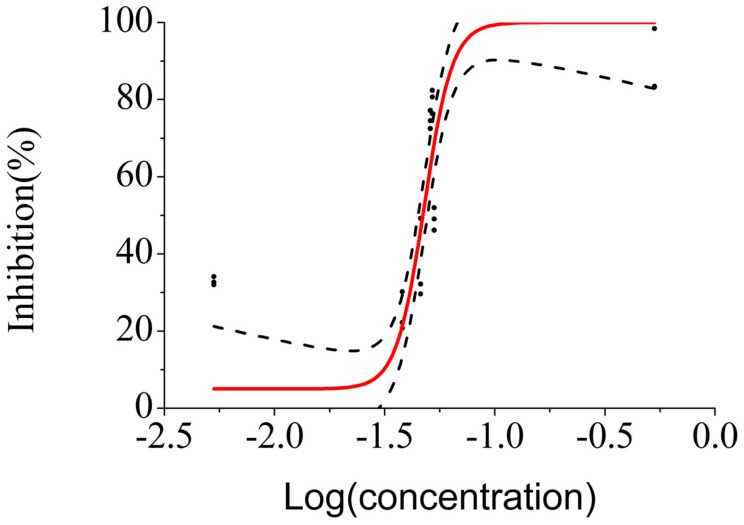
Inhibition of luminescence as a function of the logarithm of concentrations, in mg/L. Preliminary study on the sensitivity of the chip. The fitted dose–response curve is in red, and the 95% confidence intervals are displayed in black dashed lines. The experimental points are displayed in black round points.

The previous protocol was used to test the chip. Instead of using the described software, the stored results were post-processed and analyzed using Microsoft Excel and Origin Pro 8. The obtained dose response curve is presented in Figure 6. An EC50 of 6.46 × 10^−7^ M was obtained, based on the equation of the fit of the data by a logit curve, which represents a high gain of sensitivity comparing to conventional laboratorial tests. However, this result can be due to low oxygenation of the chip, so tests with different sealing films with higher air diffusivity are needed or, instead, a different approach on the material selection of the chip and respective interfaces with the opto-fluidic system.

#### 3.3.4. Comparison with Current Solutions and Possible Integration

This work studied the feasibility of a new water quality sensor for RAS, based on the record and prediction of toxicity by us of linear statistical models and a whole-cell biosensor. Over the years, the industry and academia have been focused on the use of wireless sensor nodes employing electronic and electrochemical sensors [31,32,33]. These systems focus not only in monitoring the water quality of the RAS system, but also provide control of the system during normal operation through environmental sensors and feed and also provide an alarm, if needed. Perry et al. [34] proposed a low-cost system to monitor several parameters of the fish farm facility, measuring water quality parameters such as the presence of oil, temperature, conductivity, and turbidity. Marian Barbu, Emil Ceangă, and Sergiu Caraman [35] proposed a model for monitoring and control of water quality in RAS tanks by measuring temperature, dissolved oxygen concentration, total dissolved solids, and water level in aquaculture tanks and nitrite, ammonia, water level, and nitrate in the collection tank near the biofilter. Other wireless sensor node systems also monitor salinity and water level such as the system proposed by Parra et al. [34]. They present a highly sensitive system that can monitor and control the fish farming process and can be cheaper than both our proposed solution and the solution presented by [35]. 

Nevertheless, the aforementioned systems do not give a biological response that takes in consideration the interaction between different mixture components, rather relying on threshold models which are less accurate [19]. The sensor system presented hereby is not intended to substitute environmental sensors such as temperature, pH, or dissolved oxygen, but to complement their information by collecting samples directly from the aquaculture tank, ideally from a bypassed pipe, and substitute the need for electro-chemical sensors. In future, the proposed systems will be able to alert the farmer of toxic concentrations of stressors before a problem arises. Also, it can be of assistance in control of activities such as the introduction of feed and water recirculation that can raise stressors’ concentration.

## 4. Conclusions

This work presents a new design for water quality monitoring of recirculating aquaculture systems. To the best of our knowledge, this study is the first to evaluate the interactive toxic behavior of a mixture of nitrite, un-ionized ammonia, copper, aluminum, and zinc in light of the linear concentration addition, and linear independent action models to *Aliivibrio fischeri*.

Besides, this is the first study to propose the use of a toxicology model allied with a machine learning algorithm and a whole-cell biosensor to be as sensor for RAS water quality.

A future study has to be conducted to confirm the additive behavior of a mixture of these stressors at salmon RAS limit concentrations. The study of sensitivity and accuracy of the combination of least squares regression and the LCA model to predict toxic events is to be performed as well.

Further steps including fabrication and test of the design, protocol, and associated software in laboratory and in situ are to be taken to demonstrate the use of this sensor in the RAS industry.

## Figures and Tables

**Table 1:** Maximum concentration, in mM, of each component in the mixture and total concentration of stressors in the mixture.

Ray	Nitrite (mM)	Un-Ionized Ammonia (mM)	Copper (mM)	Aluminum (mM)	Zinc (mM)	Total Concentration (mM)
1	8.99 × 10^−7^	2.32 × 10^−2^	5.25 × 10^−3^	3.60 × 10^−4^	1.22 × 10^−3^	3.00 × 10^−2^
2	1.93 × 10^−5^	3.01 × 10^−2^	6.13 × 10^−3^	1.48 × 10^−4^	9.11 × 10^−4^	3.73 × 10^−2^
3	1.48 × 10^−4^	3.74 × 10^−2^	4.91 × 10^−3^	4.41 × 10^−4^	5.70 × 10^−4^	4.35 × 10^−2^
4	7.90 × 10^−4^	1.86 × 10^−2^	5.83 × 10^−3^	2.21 × 10^−4^	1.41 × 10^−3^	2.69 × 10^−2^
5	3.66 × 10^−3^	2.67 × 10^−2^	4.44 × 10^−3^	5.39 × 10^−4^	1.06 × 10^−3^	3.64 × 10^−2^
6	1.70 × 10^−2^	3.35 × 10^−2^	5.55 × 10^−3^	2.89 × 10^−4^	7.55 × 10^−4^	5.71 × 10^−2^
7	9.04 × 10^−2^	4.21 × 10^−2^	6.48 × 10^−3^	6.72 × 10^−4^	1.64 × 10^−3^	1.41 × 10^−1^

**Table 2:** Determined EC50 concentrations of each stressors and respective Logit curve parameters.

Chemical Specie	EC50 (M)	*p*	log*X_c_*	*A_L_*	*A_H_*
Nitrite (NO_2_^−^)	3.66 × 10^−6^	0.26432	−0.77343	0	100
Ammonia (NH_3_-N)	3.35 × 10^−5^	3.741	−0.24315	0	100
Zinc	5.83 × 10^−6^	2.88562	−1.09769	0	100
Aluminum	4.41 × 10^−7^	2.00837	−1.92469	0	100
Copper	1.22 × 10^−6^	8.05861	−0.431	0	100

**Table 3:** Simple linear regression parameter for each model (*b*_0_ and *b*_1_). A model was created for each mixture ray. Pearson correlation is also shown.

Model	LIA			LCA		
Ray	*b*_0_	*b*_1_	Pearson Correlation	*b*_0_	*b*_1_	Pearson Correlation
1	1.26497	0.35818	0.98758	−20.32265	11.06486	0.98916
2	1.059	0.397	0.943	−21.28508	12.37994	0.94804
3	0.66542	0.51735	0.85178	−18.64007	11.05947	0.84929
4	2.58719	0.28146	0.79365	−13.15791	8.14889	0.79927
5	2.41787	0.39283	0.83234	−14.15261	9.11089	0.84284
6	1.80923	0.3959%	0.75313	−14.57845	10.50199	0.76132
7	−0.33947	0.60823	0.8255	−1.00294	1.15952	0.86081

**Table 4:** Estimated EC50 concentrations for the concentration addition and independent action models and the experimental values, relative deviation, and MDR for each ray

	LCA			LIA			Real
Ray	EC50 (M)	Deviation (%)	MDR	EC50 (M)	Deviation (%)	MDR	EC50 (M)
1	3.79 × 10^−3^	2.81%	1.03	5.43 × 10^−2^	193.31%	13.93	3.90 × 10^−3^
2	4.32 × 10^−3^	3.22%	0.97	8.73 × 10^−2^	1983.46%	20.83	4.19 × 10^−3^
3	3.82 × 10^−4^	99.34%	150.92	2.16 × 10^−1^	275.11%	3.75	5.76 × 10^−2^
4	1.01 × 10^−4^	99.42%	173.73	2.59 × 10^−3^	85.30%	0.15	1.76 × 10^−2^
5	6.84 × 10^−7^	100.00%	33,900.65	3.82 × 10^−3^	83.53%	0.16	2.32 × 10^−2^
6	1.31 × 10^−8^	100.00%	3,718,766.94	3.82 × 10^−3^	92.15%	0.08	4.87 × 10^−2^
7	2.00 × 10^−4^	99.86%	721.33	2.08	1342.41%	14.42	1.44 × 10^−1^

**Table 5:** Recommended limit values for fish farming of Atlantic salmon in RAS systems, both in mass and molar concentration.

Stressor	Concentration (mg/L)	Concentration (mM)
Nitrite	0.1 [4]	2.17 × 10^−3^
Un-ionized ammonia	0.030–0.146 [7]	1.76 × 10^−3^–8.57 × 10^−3^
Copper	0.0006–0.030 [2]	9.44 × 10^−6^–4.72 × 10^−4^
Aluminum	0.015–0.020 [7]	5.56 × 10^−4^–7.41 × 10^−4^
Zinc	0.053 [27]	8.11 × 10^−4^

## Supplementary Materials

The following are available online at http://www.mdpi.com/1424-8220/18/9/2848/s1, Tables S1–S4.
