# Supplementary material for: Monitoring Aquaculture Water Quality: Design of an Early Warning Sensor with Aliivibrio fischeri and Predictive Models
Source: Sensors (Basel). 2018 Aug 29;18(9):2848. doi: 10.3390/s18092848 (PMC6164392; doi:10.3390/s18092848)
Supplement: Supplementary file 1 [file sensors-18-02848-s001.pdf]

Supplementary Materials

Monitoring Aquaculture Water Quality: Design of An Early Warning Sensor with *Aliivibrio fischeri* and Predictive Models

Luís F. B. A. da Silva [1,†], Zhaochu Yang [1,†,*], Nuno M. M. Pires [1,3,†], Tao Dong [1,*], Hans-Christian Teien [3], Trond Storebakken [4] and Brit Salbu [3]

[1] Institute of Applied Micro-Nano Science and Technology - IAMNST, Chongqing Key Laboratory of Colleges and Universities on Micro-Nano Systems Technology and Smart Transducing, Chongqing Engineering Laboratory for Detection, Control and Integrated System, National Research Base of Intelligent Manufacturing Service, Chongqing Technology and Business University, Nan'an District, Chongqing 400067, China

[2] Department of Microsystems - IMS, Faculty of Technology, Natural Sciences and Maritime Sciences, University of South-Eastern Norway, Postboks 235, 3603 Kongsberg, Norway

[3] Centre for Environmental Radioactivity (CERAD CoE), Norwegian University of Life Sciences (NMBU), Faculty of Environmental Sciences and Natural Resource Management, P.O. Box 5003, NO-1432 Ås, Norway

[4] Faculty of Biosciences, Department of Animal and Aquacultural Sciences, Norwegian University of Life Sciences, P.O. Box 5003, N-1432 Ås, Norway

* Correspondence: tao.dong@usn.no (T.D.), telephone: +47-310-093-21; zhaochu.yang@ctbu.edu.cn (Z.Y.), telephone: +86 13018369264

† These authors contributed equally to this work.

Table of Contents

Table S1-Uniform table used to design each ray……………………………………………………………..2

Table S2-Concentrations of Copper used in the single tests, in mg/L…………………………………………...2

Table S3-Concentrations of Zinc used in the single tests per dilution, in mg/L……………………………….2

Table S4- Concentrations of Aluminum used in the single tests per dilution, in mg/L……………………….2

Table S1-Uniform table used to design each ray

Ray	Nitrite	Ammonia	Copper	Aluminum	Zinc
1	EC10	EC20	EC30	EC40	EC50
2	EC20	EC40	EC60	EC10	EC30
3	EC30	EC60	EC20	EC50	EC10
4	EC40	EC10	EC50	EC20	EC60
5	EC50	EC30	EC10	EC60	EC40
6	EC60	EC50	EC40	EC30	EC20
7	EC70	EC70	EC70	EC70	EC70

Table S2-Concentrations of Copper used in the single tests, in mg/L.

Dilution	Concentration (mg/L)
1	3
2	1
3	0,3
4	0,15
5	0,030
6	0,009
7	0,002

Table S3-Concentrations of Zinc used in the single tests per dilution, in mg/L.

Dilution	Aluminium concentration
1	0,3 mg/L
2	0,15 mg/L
3	0,075 mg/L
4	0,015 mg/L
5	0.0125 mg/L
6	0,01 mg/L
7	0,0015 mg/L

Table S4- Concentrations of Aluminum used in the single tests per dilution, in mg/L.

Dilution	Concentration
1	0,53 mg/L
2	0,053 mg/L
3	0,051
4	0,052mg/L
5	0,046
6	0,038 mg/L
7	0,0053 mg/L
